# Solvent‐Driven Synthesis of DNA‐Based Liquid Crystalline Organogels with Extraordinary Stretchability, Self‐Healing, and Higher‐Order Structural Assembly

**DOI:** 10.1002/smll.202500607

**Published:** 2025-03-11

**Authors:** Baekman Kim, Geonhyeong Park, Geunjung Lee, Juri Kim, Changjae Lee, Jesse G. Park, Mingeun Kim, Jin Suk Myung, Hyungju Ahn, Soon Mo Park, Woo Jin Choi, Dong Ki Yoon

**Affiliations:** ^1^ Department of Chemistry Korea Advanced Institute of Science and Technology (KAIST) Daejeon 34141 Republic of Korea; ^2^ Chemical Materials Solutions Center Korea Research Institute of Chemical Technology (KRICT) Daejeon 34114 Republic of Korea; ^3^ Pohang Accelerator Laboratory (PAL) Pohang 37673 Republic of Korea; ^4^ Department of Chemical and Biomolecular Engineering Cornell University Ithaca 14853 USA

**Keywords:** cationic surfactant, DNA, liquid crystal, organogel, rheology

## Abstract

The fabrication of liquid crystalline (LC) organogel via supramolecular interactions between Deoxyribonucleic acid (DNA) and lyotropic cationic surfactant containing cyanobiphenyl moiety is reported. The fabricated organogel endows dominantly viscous behavior in dimethyl sulfoxide (DMSO) and elastic behavior in n‐propanol (n‐PrOH), respectively. By judiciously controlling the viscosity, DMSO organogels can be drawn to form a fiber with an elongation of up to 4.6 × 10^3^%, emphasizing extraordinary stretchability. Higher‐order structures, such as yarn and a co‐alignment matrix for anisotropic particles, can be produced by assembling a single fiber. On the other hand, free‐standing n‐PrOH organogels demonstrate a remarkable storage modulus of 10^5^ Pa and manifest self‐healing properties. Finally, a sustainable method by transforming n‐PrOH gel into an aerogel through critical point drying (CPD), enabling its use as an adsorbent while simultaneously enhancing its reusability is proposed. It is envisaged that these DNA‐based organogels, through conceivable combinations between DNA as a building block and cationic surfactant with functionalities as a counterpart, will contribute to significant progress in DNA‐based multi‐functional organogels.

## Graphical abstract

The dual mechanical behaviors of liquid crystalline (LC) organogels via supramolecular interactions between Deoxyribonucleic acid (DNA) and a lyotropic cationic surfactant containing a cyanobiphenyl moiety is demonstrated. Fabricated organogel displays solvent‐dependent viscoelastic properties and presents promising applications as a fiber, yarn, bioprinting, self‐healing gel, and responsive biomaterial. This strategy highlights the programmability of DNA‐based organogels via judicious control of organic solvents and provides promising applications in multiple functional materials.

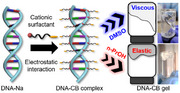

## 1 Introduction

Deoxyribonucleic acid (DNA) is an intensely studied functional building block that is massively produced and present in nature.^[^ ^1^, ^2^ ^]^ DNA is a semi‐flexible polymer with controlled contour and persistence lengths, allowing for consistent elastic properties that satisfy the Onsager theory that the lyotropic liquid crystal (LC) phase is induced above specific concentrations.^[^ ^3^, ^4^ ^]^ Owing to LC's collective behavior, DNA orientation can be manipulated through evaporation‐induced self‐assembly (EISA), shearing, or with the help of topographic control;^[^ ^5^, ^6^, ^7^, ^8^, ^9^, ^10^, ^11^, ^12^, ^13^, ^14^, ^15^ ^]^ Moreover, DNA possesses a phosphate ion which contributes to an overall negative charge, thereby making DNA an attractive anionic building block.^[^ ^16^ ^]^ More specifically, the linear charge density of double‐stranded DNA (dsDNA) is 0.59 charges Å^−1^ and the charge density per unit area reaches 253 mC cm^−2^. These values represent that dsDNA are enormously charged biomaterials which far outweighs other charged biomaterials such as cellulose nanocrystals by 20 000 times. This implies DNA can bind with other cations or metal ions more efficiently, thereby inducing DNA‐based functional materials with enhanced properties.

Until now, DNA has found potential applications as DNA‐based gels. Conventionally, many studies have been focused on DNA‐based hydrogels, which have grabbed significant interest due to their high water retention capacity and biocompatibility, making them a compelling candidate for biomedical applications such as drug delivery, skin patches, and hemostasis.^[^ ^17^, ^18^, ^19^, ^20^, ^21^ ^]^ Recent research has extended the scope of DNA‐based materials beyond hydrogels, leveraging DNA's unique properties such as molecular recognition and lyotropic phase behavior. DNA organogels offer enhanced solvent compatibility and tunable mechanical properties which broadens DNA organogel's applications. Indeed, DNA organogels offer a higher degree of programmability. DNA possesses superior molecular recognition through the integration of desirable functionalities via electrostatic interactions and hydrogen bonding.^[^ ^22^, ^23^, ^24^, ^25^, ^26^ ^]^ Through secondary interactions, DNA can easily interact with surfactants, metal ions, or additives which endow DNA organogel with desirable properties in a controlled manner. Simultaneously, facile control over solvent selectivity also offers a level of tunability that is difficult to achieve with conventional DNA hydrogels. Indeed, different solvents exhibit distinct physicochemical properties such as viscosity, solubility, swelling ratio, boiling point, and freezing point. These diverse characteristics enable the design of programmable gels with tailored functionalities. In the past, there have been several organogel studies that have utilized the unique properties of solvents and further extended to applications.^[^ ^27^ ^]^ For instance, organogels have adopted dimethyl sulfoxide (DMSO) and thus, organogels can overcome evaporation‐induced structure collapse. Similarly, hydrocarbon or alcohol solvents such as hexane and n‐propanol (n‐PrOH) can be introduced. In doing so, concerns regarding freezing can be resolved as hydrocarbon or alcohol solvents can remain intact at temperatures below 0 °C. Unlike hydrogels which become brittle and ice‐like solids that fracture easily at subzero temperatures, previous reports assert that organogels (n‐dodecane as a solvent) maintain mechanical stability at −10 °C.^[^ ^28^ ^]^ Additionally, the intrinsic properties of solvents such as hydrophobicity, hydrophilicity, and omniphobicity can be harnessed for anti‐fouling and anti‐adhesion applications. Jiang et al. previously reported the fabrication of organogels with remarkable self‐replenishing anti‐waxing properties.^[^ ^29^ ^]^ In their study, a PDMS network infused with crude oil effectively reduced paraffin wax adhesion to the organogel surface. Low adhesion was attributed to the infiltration of oil into the PDMS network, where the oil acted as a lubricant thereby minimizing solid/solid contact and adhesion. Last but not least, Smalyukh et al. fabricated ordered nematic liquid crystal (LC) gel films using cellulose nanofibers (CNFs) and 5CB, a thermotropic LC fluid.^[^ ^30^ ^]^ When the temperature exceeded 35 °C which is the nematic‐isotropic transition temperature (T_NI_) of 5CB, the orientational ordering of CNFs within the gel network changed. In turn, this leads to changes in both birefringence and transmittance of gel films. This research suggested potential applications of organogel for flexible displays and smart windows. Overall, on‐demand solvent selection would allow the fabrication of versatile organogels for various applications.

Previously, Liu et al. proposed the fabrication of DNA‐based organogels through complexation of DNA with aliphatic cationic surfactants such as cetyltrimethylammonium bromide (CTAB), didodecyldimethylammonium bromide (DDAB), and didecyldimethylammonium bromide (DEAB).^[^ ^31^ ^]^ By deliberately controlling the cationic source and organic solvent, it is possible to endow DNA‐based organogel with desired functionalities. It is reported that remarkable mechanical properties arise from forming LC ordering within DNA‐based organogels. Extrapolating from the previous report, where the formation of the LC phase plays a significant influence on the mechanical properties, we conjecture that using an LC molecule which is an aromatic cationic surfactant with a rigid biphenyl moiety and flexible alkyl chains that resemble 4‐Cyano‐4′‐pentylbiphenyl (5CB) will induce the LC phase. As a result, precise control over anisotropic properties, such as tunable mechanical and optical functionalities in DNA organogel is targeted. Herein, we report on DNA‐based organogel fabrication through electrostatic complexation between DNA and cationic surfactant containing cyanobiphenyl moiety. The DNA‐based organogels display distinct rheological properties depending on the choices of solvent (DMSO or n‐PrOH), which are examined using polarized optical microscopy (POM), scanning electron microscopy (SEM), small/wide angle X‐ray scattering (SAXS/WAXS), tensile test, and rheometer. DMSO gel transforms into a µm‐scale thin fiber upon strain, representing outstanding processability and stretchability. The promising applications of fiber as a co‐alignment matrix are verified by adding anisotropic particles, such as a metal‐organic framework (MOF) to show linear fluorescence and iron oxide particles to show magnetic‐responsive behavior. Besides, n‐PrOH gel manifests self‐healing properties in which restoration of mechanical property, as well as molecular alignment, are witnessed. Finally, we propose a future perspective of organogel by transforming it into an aerogel via critical point drying (CPD). Hence, our method suggests a facile method for fabricating DNA‐based LC organogel, which imparts tunable properties and applications by varying solvents.

## 2 Results and Discussions

**Figure 1:**
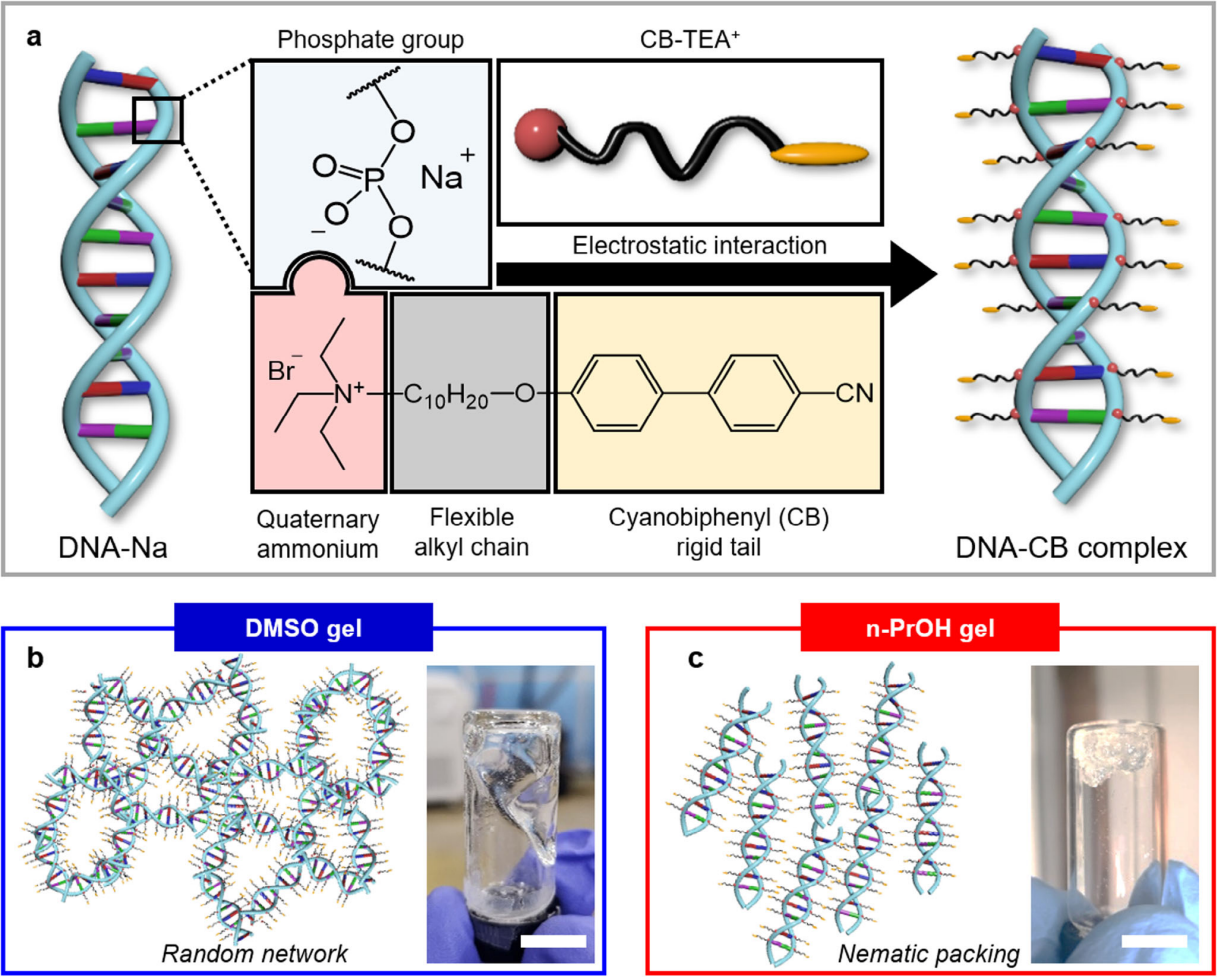
Fabrication of DNA‐CB complex and DNA‐CB orgenogel. a) DNA‐CB complex is synthesized through electrostatic attraction between DNA‐Na and cationic surfactant, CB‐TEA^+^. b,c) Fabrication of DMSO and n‐PrOH gel via organo‐gelation of DNA‐CB complex, respectively. A visual illustration of a molecular network within DMSO and n‐PrOH gel is illustrated.

The DNA material utilized in this research was a salmon sperm DNA duplex (Sigma–Aldrich) exhibiting a Gaussian distribution in base pairs with the center point of 2000 base pairs. The contour length of 680 nm. This DNA is considered a semi‐flexible polymer with a persistence length (*p*) of ≈50 nm and an average molecular weight is 1300 kDa.^[^ ^10^, ^32^ ^]^ To aqueous DNA solution, synthesized triethylammoniodecyloxy cyanobiphenyl (CB‐TEA) with bromide as a counterion is added. The reaction is carried out at a stoichiometric ratio of 1:1. Upon vigorously mixing, the DNA‐CB complex is precipitated due to complexation via electrostatic interaction between phosphate groups of DNA and quaternary amine of CB‐TEA (**Figure** **1**a). Elemental analysis (EA) confirmed that CB ligands fully cover the DNA molecules (Figure S9, Supporting Information). Detailed information regarding synthesis is included in the Experimental Section.

**Figure 2:**
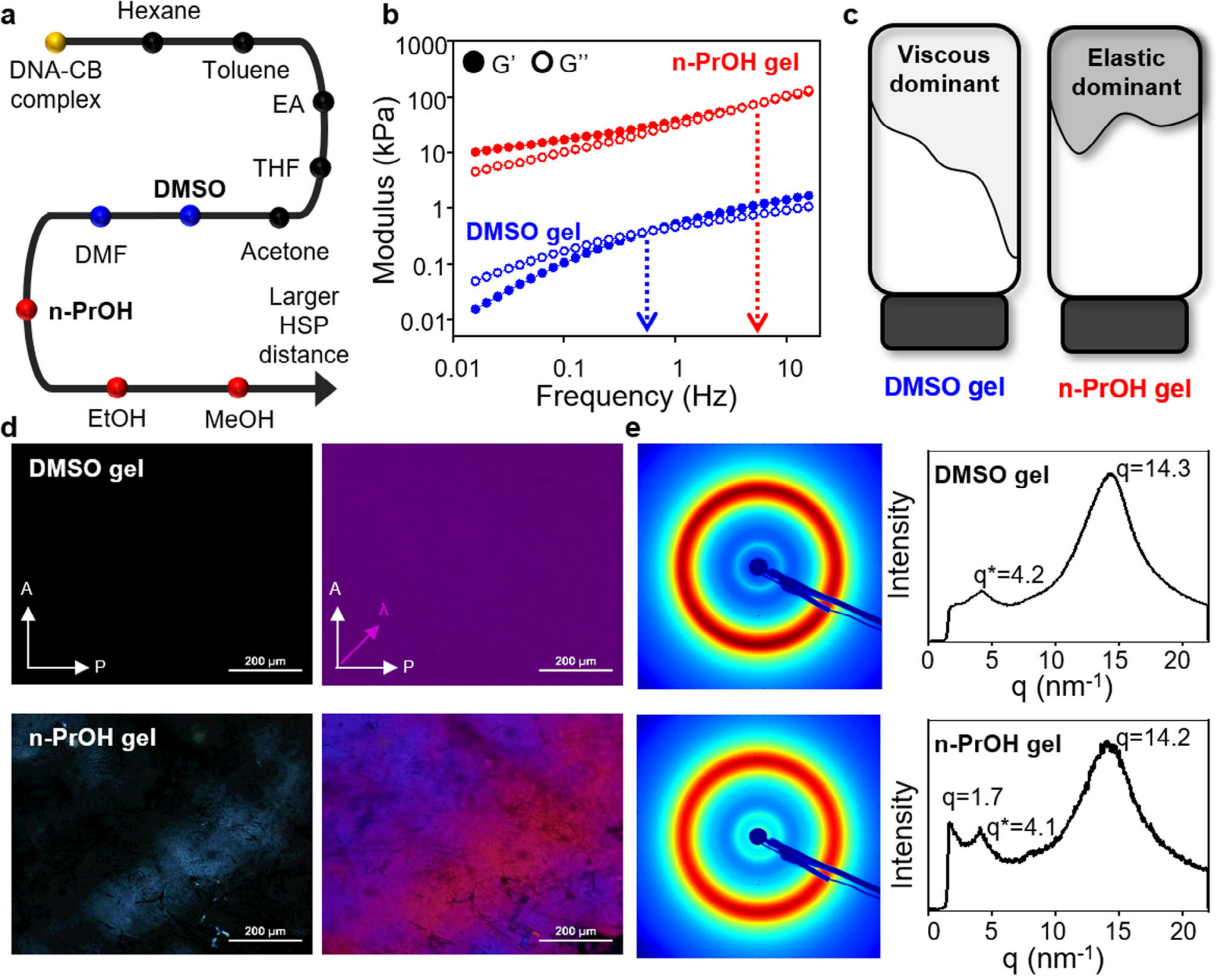
Characterization of fabricated DMSO and n‐PrOH gel, respectively. a) Solvent compatibility of DNA‐CB complex calculated by Hansen solubility parameter (HSP). The larger the HSP distance, the less favorable the interaction. The black color indicates no reaction between the complex and solvent. b, c) Comparison of viscoelastic behavior of DMSO and n‐PrOH gel, respectively. DMSO gel demonstrates viscous‐dominant features, representing more liquid‐like properties. In contrast, n‐PrOH gel displays elastic‐dominant behavior, illustrating a solid‐like response. The schematic highlights the dominant rheological properties in each gel. d) POM images of DMSO and n‐PrOH gel, respectively. DMSO gel shows an isotropic phase whereas n‐PrOH gel demonstrates birefringent textures, indicating an anisotropic nature. e) 2D SAXS patterns and corresponding 1D intensity profiles of DMSO and n‐PrOH gels, respectively. The DMSO gel shows a peak at q = 14.3 nm^−1^ which represents π–π stacking. In contrast, n‐PrOH gel possesses additional peaks at q = 1.7 nm^−1^ which represent a nematic peak with a periodic distance of ≈3.7 nm, suggesting a more ordered molecular arrangement. Peak at q ≈ 4 nm^−1^ comes from a Kapton tape. The scale bars are d) 200 µm (POM images).

After centrifuge and removal of the supernatant, the DNA‐CB complex is taken and used for organo‐gelation (Figure 1b,c). The synthesized DNA‐CB complex demonstrates distinct behavior upon contact with various organic solvents. To investigate the effect of solvents on the DNA‐CB complex, Hansen solubility parameter (HSP) calculations are performed for both the DNA‐CB complex and the organic solvents (**Figure** **2**a; Figure S10, Supporting Information). Regarding the selection of DMSO, HSP values indicate that DMSO has a stronger polymer‐solvent interaction than DMF, making it a more suitable choice. Additionally, DMSO was preferred over DMF due to compatibility and safety concerns. Indeed, DMF was avoided because it is highly toxic, making it unsuitable for both fabrication and practical applications. On the other hand, DMSO is frequently used in various applications upon proper treatment such as dilution and in the form of DMSO/H_2_O bigel.^[^ ^33^ ^]^ Among alcohol‐based solvents such as ethanol, methanol, and n‐propanol, n‐ProH was found to be the most suitable choice. n‐ProH possesses the most favorable HSP value which ensures enhanced compatibility with the polymer. In addition, n‐ProH possesses the highest boiling point of 97 °C which curbs rapid solvent evaporation at room temperature. Therefore, the gel's structural integrity was maintained throughout the experiment and analysis. DNA‐CB complex in DMSO exhibits a viscous behavior and it slowly flows along the walls of the vial, as can be observed by the naked eye. In contrast, the DNA‐CB complex in n‐PrOH demonstrates elastic behavior, and it maintains its original shape even after the vial is tilted. The primary reason for this phenomenon is that the CB ligand predominantly interacts with the organic solvent, whereas the DNA backbone hardly interacts with the organic solvent. This implies that the CB ligand is primarily responsible for determining the entire DNA‐CB complex's interaction behavior with organic solvents. Indeed, the CB ligand is soluble in DMSO, thereby inducing the entire DNA‐CB complex soluble in DMSO. In contrast, n‐ProH exhibits a different reaction behavior where swelling of an organogel is induced. These findings highlight how the DNA‐CB complex responds differently to various organic solvents, providing insight into their potential for forming programmable organogels.

The viscoelastic properties of the two organogels are studied by rheometer (Figure 2b,c). Indeed, the crossover point at which the storage modulus (G') and the loss modulus (G″) become equal exists in both gels. In DMSO gel, loss modulus (G″) surpasses storage modulus (G′) at low frequency, and this trend is reversed as frequency increases. Since loss modulus (G″) demonstrates how much energy is lost during deformation, a higher value means DMSO gel predominantly possesses energy‐dissipating properties, thereby signifying that liquid‐like properties are more dominant. In stark contrast, storage modulus (G′) outweighs loss modulus (G″) at low frequency in n‐PrOH gel, representing solid‐like properties that are more dominant in n‐PrOH gel. Further analysis by POM supports the liquid‐like (isotropic) phase of DMSO gel (Figure 2d). Due to the absence of anisotropy in liquid, black and purple images are obtained in POM images of DMSO gel. Unlike DMSO gel, birefringence is found in POM images, confirming high packing density and molecular alignment within n‐PrOH gel. This is because birefringence occurs as light passes through anisotropic materials such as aligned molecules and undergoes refraction. DNA‐CB complex forms various structures ranging from nematic packing to random networks depending on the type of solvents. To validate this statement, analysis of the internal structure is determined through XRD (Figure 2e). Here, the peak at *q* = 4.1–4.2 nm^−1^ is due to the Kapton tape as a reference (Figure S12, Supporting Information). Two peaks corresponding to 0.41 Å^−1^ and 0.82 Å^−1^ were observed which exhibit a twofold difference, indicating that 0.82 is an integer multiple of 0.41. According to Bragg's law, the diffraction order, n, equals 2 (Equation 1). *λ* represents the wavelength of the incident X‐ray beam, *d* represents the interplanar spacing between atomic layers in a crystalline material, and 𝜃 represents the angle between the incident X‐ray beam and the crystal planes.

(1) nλ=2dsinθ

In n‐PrOH gel, the *q =* 1.7 nm^−1^ corresponds to nematic packing, and the formed nematic phase demonstrates an average distance of 3.69 nm. Previous reports state the presence of nematic LC mesophase of DNA‐DDAB organogel by demonstrating peaks at *q* = 1.47 nm^−1^ in a SAXS profile. In contrast, a ring pattern is witnessed in DMSO gel. No specific molecular arrangement beyond the π–π interaction (*q* = 14.3 nm^−1^) is detected in the internal structure according to *q* value analysis. At this point, further internal structure analysis by dynamic light scattering (DLS) reveals a random polymer network that resembles aggregation with a hydrodynamic diameter (D_h_) of 185 nm formed in DMSO gel (Figure S13, Supporting Information). Indeed, Li et al. explain the mechanism for forming an aggregate when cations bind to DNA segments.^[^ ^19^ ^]^ These interactions induce condensation of DNA and cations into a core aggregate. Indeed, further analysis by cryoTEM and DLS supports the formation of core aggregate. Based on the aforementioned rheometer, POM, and XRD analysis, the visualization of the internal structure for both gels is illustrated in Figure 1b,c.

**Figure 3:**
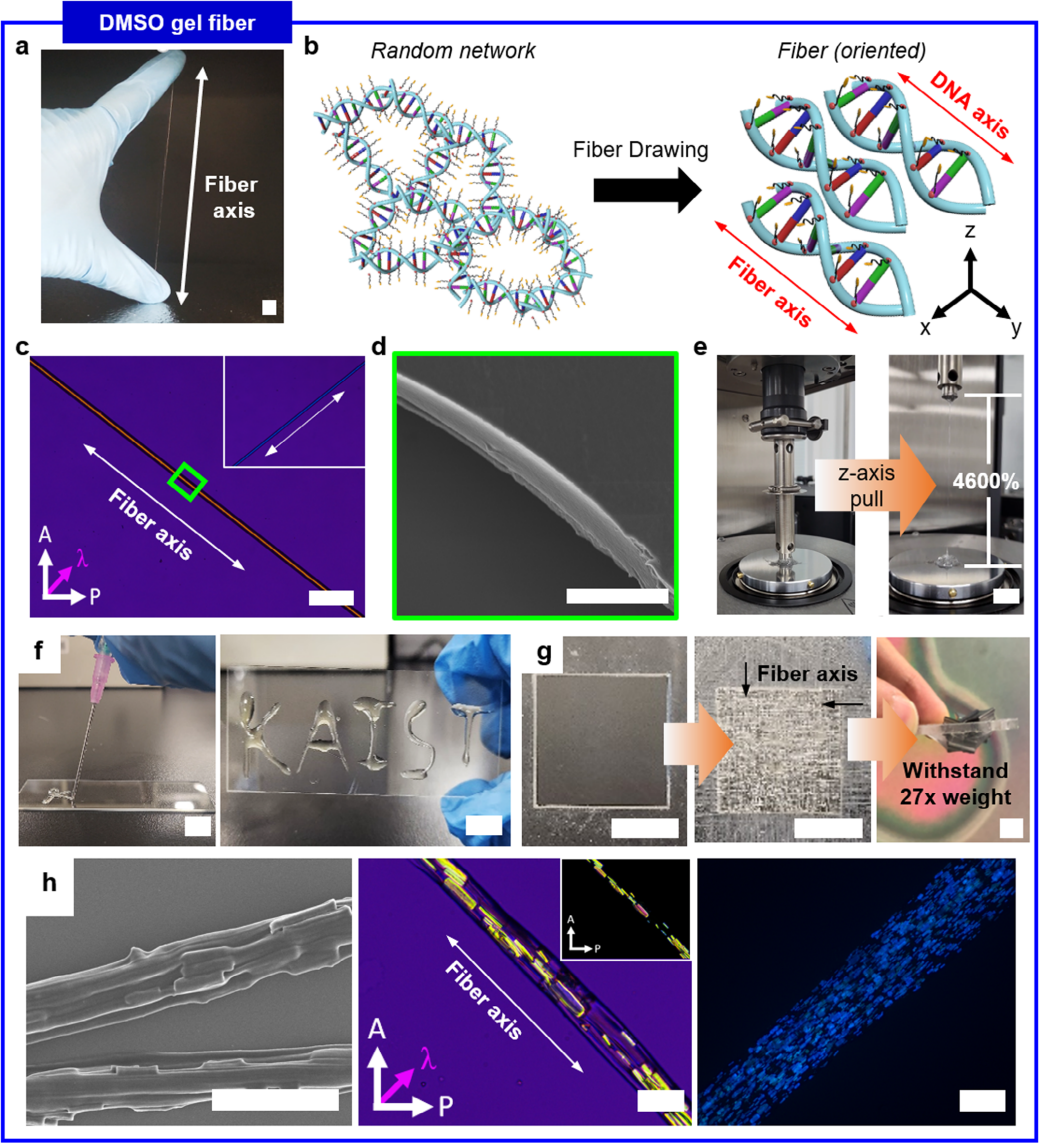
Fabrication and characterization of DMSO gel fiber. a) Illustration showing that highly aligned polymer fibers can be drawn by simply pulling DMSO gel. b) Illustration of a change in orientation of DMSO gel upon drawing a fiber. The orientation of surfactant with respect to DNA backbone changes upon fiber drawing, thereby inducing a positive birefringence. c, d) POM images of a drawn fiber and SEM images illustrating the magnification of a drawn fiber. e) z‐axis pull test of DMSO gel fiber, representing exceptional stretchability. f) printing application via extrusion of low viscosity (10–20 w/v%) DMSO gel. g) Fabrication of yarn through template‐assisted interweaving of DMSO gel with high viscosity (30–50 w/v%). The fabricated yarn can hold up to several gram‐scale. h) Co‐alignment of anisotropic metal‐organic frameworks (MOFs) via DNA‐TEA organogel matrix. The scale bars are a) 1 cm; c) 50 µm (POM images); d) 5 µm (SEM image); e) 1 cm; f) 1 cm; g) 1 cm; h) 50 µm (SEM image), 10 µm (POM image), respectively.

In DMSO gel, the viscosity can be manipulated by varying concentrations of DNA‐CB complex (Figure S14, Supporting Information). Indeed, results confirm the proportional relationship between DNA‐CB complex concentration and viscosity. Interestingly, DMSO gel demonstrates shear thinning behavior. Indeed, deformation causes change in rheological behavior and previous reports suggest that shear thinning is due to the weakening of binding interactions by shear stress. By utilizing the shear thinning behavior, the fiber extrusion is viable. Simply pulling DMSO gel allows highly aligned polymer fibers to be drawn with remarkable stretchability (**Figure** **3**a). Figure 3b displays the formation of an orientation from a random polymer network upon fiber extrusion. Subsequently, the drawn fiber is analyzed through POM. The crossed polarizers generate the purple color in the background. We investigate the internal structure of aligned DMSO gel using POM with the retardation plate inserted. Initially, the previously reported aligned DNA demonstrates negative birefringence, therefore blue color is obtained when DNA is aligned perpendicular to the axis of the retardation plate and yellow color is obtained when DNA is aligned parallel to the axis of the retardation plate.^[^ ^10^, ^34^ ^]^ Similarly, synthesized CB ligands also possess negative birefringence as well (Figure S5, Supporting Information).^[^ ^35^ ^]^ Nevertheless, the color of birefringence is yellow when the DNA‐CB gel fiber is aligned perpendicular to the axis of the retardation plate under the POM (Figure 3c). The result indicates that DNA‐CB gel fiber possesses positive birefringence which does not match with the previously reported color of aligned DNA nor CB ligand. This leads to the conclusion that a change in orientation occurs when DNA and the CB ligand interact, and the birefringence pattern is reversed. The alignment of the CB ligand in DMSO gel fiber is confirmed by polarized FT–IR data (Figure S16, Supporting Information). From the polarized FT–IR data, maximum absorption intensity at 105° is obtained which indicates that the CB ligand adopts orientation with 105° tilted in the *z‐*axis, whereas the DNA backbone lies in the *xy*‐axis (Figure 3b; Figure S16). Moreover, the birefringence of the CB ligand is more dominant than DNA in the DNA‐CB complex because the birefringence is significantly stronger in cyanobiphenyl moiety than in DNA. Consequently, the origin of birefringence in CB ligand and DNA competes as CB ligand and DNA are no longer parallel, rather orients in a different plane. Next, uniaxial fiber with a dimension of a few micrometers is fabricated and successful alignment is further confirmed by SEM data (Figure 3d), indeed, XRD analysis unequivocally corroborates the highly oriented molecules within the fiber with an order parameter (***S***) of 0.32 (Figure S15, Supporting Information). Perfectly aligned particles display *S*  =  1, while randomly oriented particles have *S*  =  0. In a previous study, Ahn et al. reported the fabrication of liquid crystalline fiber via UV‐assisted melt spinning and proposed promising applications as a smart textile and clothing.^[^ ^36^ ^]^ Likewise, the reported order parameter value of 0.37 was yielded under the nematic phase. This further validates that an order parameter of 0.32 signifies fibers with orientational ordering, More quantitatively, the z‐axis pulling test on fiber is conducted and halts upon reaching the limit of the equipment (≈4600%) (Figure 3e), indicating extraordinary stretchability. One particularly intriguing result is that DNA‐CB gel fiber also displays lyotropic behavior. This is evident from the observation that birefringence is not immediately observed after extrusion. However, as DMSO evaporates gradually upon exposure to air, birefringence progressively develops. This phenomenon suggests that the increasing concentration of DNA‐CB leads to the emergence of birefringence, which is a characteristic feature of lyotropic liquid crystals. This behavior arises due to the molecular rearrangement that occurs as DNA‐CB concentration increases, facilitating birefringent structures. Consequently, the lyotropic nature of DNA‐CB gel fibers are attributable to the interaction between the lyotropic properties of DNA and the CB surfactants used in the system.

**Figure 4:**
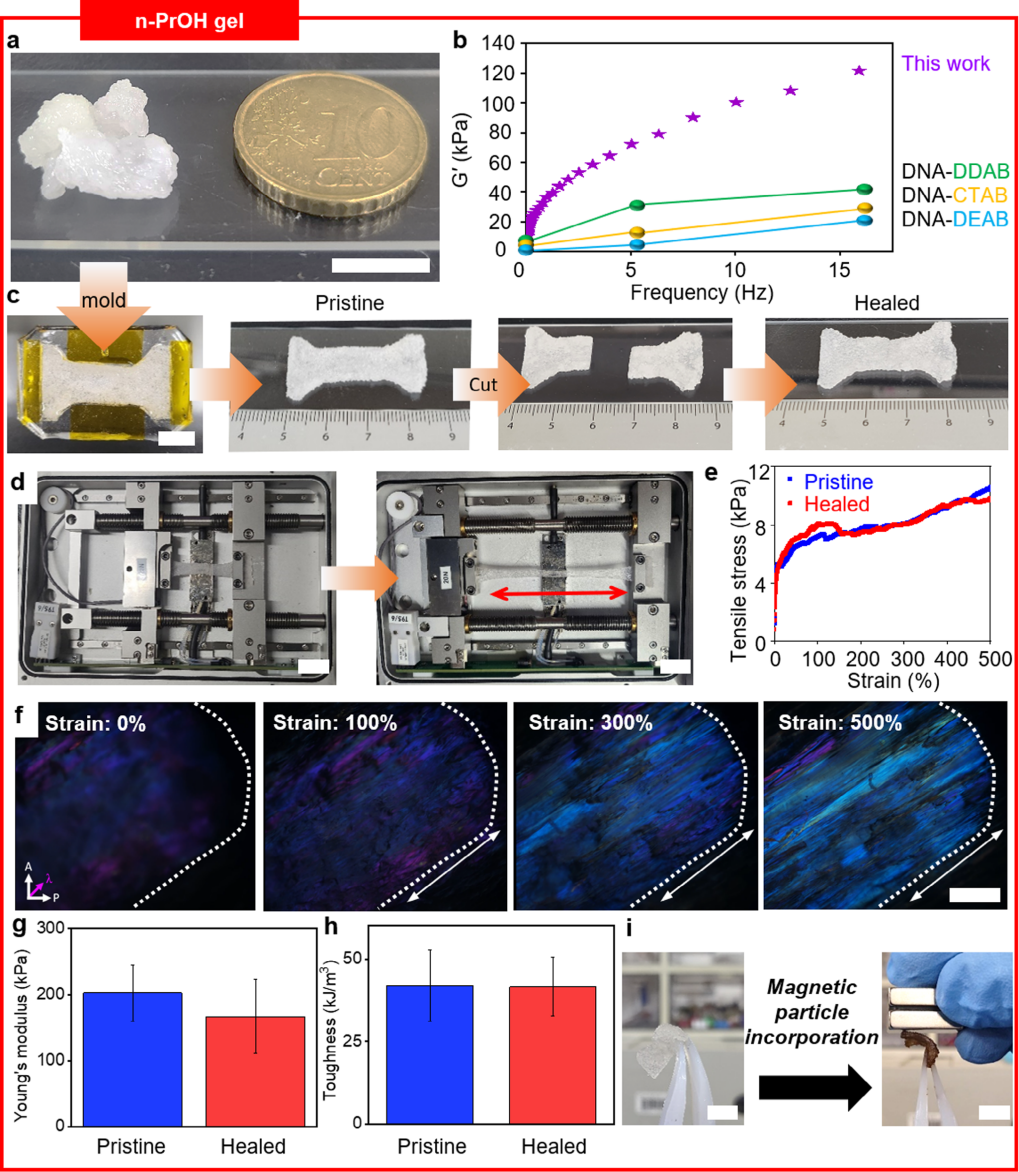
Fabrication and characterization of n‐PrOH gel. a) Illustration of n‐PrOH gel after solvent impregnation of DNA‐CB complex in n‐PrOH. b) Dynamic mechanical analysis of n‐PrOH gels using a shear rheometer and comparison with other DNA‐cationic surfactant organogel previously reported in the literature. Chemical structures of surfactants are demonstrated, respectively. c) Shape molding process of n‐PrOH gel into a dog‐bone shape. d) Visual illustration of a tensile test, indicating high stretchability of n‐PrOH gel. e) Analysis of tensile stress between pristine and healed n‐PrOH gel, representing superb self‐healing properties. f) POM images of n‐PrOH gel with changes in strain during tensile test. g,h) Young's modulus and toughness of n‐PrOH gel. i) Illustration of magnetic‐responsive behavior of n‐PrOH gel after incorporation of Fe_3_O_4_ particle. The scale bars are a) 1 cm; c) 1 cm; d) 1 cm; f) 500 µm (POM images); i) 1 cm, respectively.

Next, we delve into appealing applications of DMSO gel fiber, expanding its roles in material science. On the one hand, by judiciously controlling the viscosity, DMSO gel finds practical applications in patterning, fiber, and yarn (Figure 3f,g). Indeed, DMSO gel with a viscosity lower than 20 w/v% can be applied for patterning to generate a desired shape. For instance, DMSO gel with low viscosity can be extruded from a needle gauge to pattern KAIST on a glass substrate, indicating application as an ink for printing. Similarly, DMSO gel with a higher viscosity of more than 30 w/v% allows yarn fabrication to be feasible. By interweaving fibers perpendicularly in a mold, it is possible to produce a robust yarn that can withstand up to several gram‐scale. Furthermore, the co‐alignment of various anisotropic materials such as gold nanorods (AuNRs) and metal‐organic framework (MOF) crystals is viable to endow unique properties. Previously, Ahn et al. achieved DNA‐guided organic semiconductors by incorporating tri(8‐hydroxyquinoline) aluminum (Alq_3_) rods.^[^ ^37^ ^]^ Prolonged fluorescence lifetime and increased luminescent response by 1.6‐fold were witnessed. Consequently, this opens a new avenue for OLED materials, introducing novel photonic functions. Moreover, previous reports also utilized DNA as a matrix to align the water‐soluble conjugated polymer, poly[3‐(potassium‐7‐hexanoate)‐thiophene‐2,5‐diyl] (P3PHT), in organic field‐effect transistors (OFETs).^[^ ^38^ ^]^ The P3PHT/DNA blend was effectively aligned using a simple shearing method, significantly enhancing charge carrier mobility by improving charge transport. Additionally, copper‐mediated interactions with DNA enabled p‐doping which leads to the highest reported hole mobility for water‐soluble polythiophenes. The integration of DNA nanotechnology in organic devices as high‐performance organic optoelectronic systems is suggested as a promising application. In our experiment, MOF crystals are incorporated within a gel fiber. MOFs possess well‐defined structures and tunable conductivity, owing to the selective choice of metal ions and organic ligands.^[^ ^39^, ^40^, ^41^, ^42^ ^]^ Since MOFs possess anisotropic crystal structures, they allow directional charge transport. In other words, it is possible to control the alignment and orientation of MOF crystals, thus inducing varying electrical and optical properties. As a result, precise control of orientation is crucial for advancing their practical applications. Here, NU‐1000 crystals act as an anisotropic guest material and DMSO gel fiber acts as an alignment matrix. Indeed, SEM and POM images tangibly denote the incorporation of MOF crystals within a highly aligned fiber that would otherwise be distributed in an anisotropic manner (Figure 3h). Additionally, the presence of a fluorescent organic linker in NU‐1000 induces strong fluorescence intensities within a fiber in fluorescence microscope images. These results imply the potential research of anisotropic particles‐incorporated gel fiber.

n‐PrOH gel is fabricated by simple solvent impregnation of DNA‐CB complex in n‐PrOH. **Figure** **4**a demonstrates the image of the n‐PrOH gel. Next, a thorough investigation of n‐PrOH gel's mechanical properties is conducted by dynamic mechanical analysis (DMA) using a shear rheometer. Remarkably, the obtained storage modulus (G′) of 120 kPa in n‐PrOH gel far outweighs previously synthesized DNA‐cationic surfactant organogel (Figure 4b). This is ascribed to the molecular design of cationic surfactants. Unlike aliphatic cationic surfactants, which only contribute to van der Waals force, the inclusion of biphenyl moiety contributes to π–π stacking. Therefore, additional secondary interactions lead to enhanced rheological properties. Indeed, previous reports substantiate the assertion by stating that adding an aromatic ring led to a similar effect as increasing the chain length.^[^ ^25^, ^26^ ^]^ Considering that the length of the surfactant alkyl chain is directly proportional to van der Waals force and thereby affects storage modulus, n‐PrOH gel's superb storage modulus indeed underpins the advantageous role of cyanobiphenyl moiety. Moreover, n‐PrOH gel also exhibits a high loss modulus (G″) which represents strong viscous properties of DNA organogel (Figure S11, Supporting Information). Thus, while a high storage modulus indicates a large amount of energy stored during deformation, a high loss modulus also represents that a significant amount of energy is dissipated during deformation. This behavior is beneficial for damping applications, where energy dissipation is desirable.

To further accentuate the novelty of n‐PrOH organogel, not only the restoration of mechanical properties but also the restoration of birefringence is verified. Through molding, n‐PrOH gel can undergo facile change into a dog‐bone shape. In addition, we tested the self‐healing properties of n‐PrOH gels (Figure 4c). Pristine organogel is severed by halves and a drop of n‐PrOH is added to the interface. Then, the severed organogel turns seamless, and recombinant organogel is achieved within a minute. Furthermore, the mechanical properties of the damaged gel are similar to the original gel regarding Young's modulus and toughness (Figure 4d–h). Previously, similar studies on DNA gel fabrication have also reported comparable values after severed gels underwent healing.^[^ ^43^ ^]^ The rapid self‐healing of the gel is facilitated by several factors including spontaneous intermolecular interactions, such as hydrophobic interactions at the rejoined surface. Moreover, solvent‐assisted diffusion fosters a liquid‐like property such that facile percolation between gels is achieved. As a result, the mechanical resilience of DNA‐based organogel is confirmed. Furthermore, both pristine and healed n‐PrOH organogel demonstrate restoration of birefringence which proves reversibility of molecular alignment upon external forces. The movies further supplement the real‐time observation of pristine and healed n‐PrOH organogel's birefringence during tensile testing. Initially, weak birefringence is observed with purple color as a background. Upon applying tension, strong positive birefringence begins to emerge in the pristine gel, signifying that molecular alignment is manifested upon shear stress (Movie S1, Supporting Information). Once the tension is removed, weak birefringence with purple color as a background reappears, indicating the diminishing of molecular alignment as witnessed in POM images of pristine n‐PrOH gel in Figure 2d. Similarly this phenomenon of birefringence disappearing and reappearing is also observed in healed gels, indicating that mechanical fidelity and birefringence restoration are preserved (Movie S2, Supporting Information). Otherwise, insufficient self‐healing would lead to the n‐PrOH gel being torn apart upon stress such that the restoration of birefringence would not be anticipated. Similar to the incorporation of anisotropic MOF crystals into the DMSO gel's matrix to achieve co‐alignment, we incorporate stimuli‐responsive particles into the n‐PrOH gel matrix to endow responsive functions. For instance, Fe_3_O_4_ magnetic particles impart magnetic‐responsive properties in n‐PrOH gel. Theoretically, n‐PrOH gel does not respond to magnets. Adding 1 mg of Fe_3_O_4_ magnetic particles into the n‐PrOH gel creates magnetic‐responsive properties (Figure 4i and Movie S3, Supporting Information). Likewise, this versatility in incorporating functional particles is anticipated to facilitate further studies on stimuli‐responsive materials and multifunctional systems.

In order to appeal to a broader interest, we focused on the perspective of n‐PrOH gel to aerogels via critical point drying (CPD). Previously, organogel was disposed of after use. Nevertheless, through this CPD process, used organogel is no longer wasted. Instead, they are transformed into an aerogel (Figure S17a). This process renders material recycling and reusability, providing an environmentally sustainable method by reducing waste and minimizing the consumption of hazardous chemicals. By enhancing fabrication efficiency, this approach facilitates mass production while maintaining sustainability and expanding the material's functional versatility. Fabricated aerogel is examined by SEM (Figure S17b). Lyophilized gels possess microporous nanostructures with an average pore size of 3 µm. This originates from a solvent exchange between n‐PrOH and carbon dioxide to generate vacancy. In the past, aerogels have garnered remarkable attention due to their potential to be adsorbents.^[^ ^44^, ^45^, ^46^ ^]^ This is ascribed to aerogel's innate porous structure, high surface area, and low density. Hence, to rationalize the state of the art, UV–vis analysis on time‐dependent organic dye adsorption is conducted (Figure S17c). Upon soaking an aerogel in crystal violet dye in DCM, an absorbance tapers off. Indeed, as an aerogel absorbs organic dye, the overall concentration of a dye continues to decrease and eventually results in an 80% decline in absorbance. The change in color of an aerogel after dye removal is illustrated (Figure S17d, Supporting Information). Moreover, the quantitative time‐dependent dye adsorption capacity of aerogel is explored. Over time, the Q_t_ value gradually increases and saturates at 0.42 mg g^−1^ (Figure S18, Supporting Information). Consequently, the aforementioned outcomes signify the application of aerogel as a sorbent, thereby enabling the removal of organic pollutants. By transforming into an aerogel, cost‐efficiency and sustainability becomes viable.

## 3 Conclusion

To summarize, we have developed a series of LC organogels ranging from viscous to elastic using DNA and cationic surfactant containing cyanobiphenyl moiety. Versatility in simultaneously transforming into fiber and gel highlights remarkable stretchability and a high degree of molecular alignment and underlines self‐healing properties and outstanding storage modulus. We believe that through combinations of stimuli‐responsive and rich chemical functionalitycontaining cationic surfactants with DNA, progress in DNA‐based multi‐functional organogels will be foreseeable.

## 4 Experimental Section

### Materials

4‐cyano‐4′‐hydroxybiphenyl and 1,10‐dibromodecane were purchased from TCI chemicals. Potassium carbonate was purchased from Alfa Aesar. Potassium iodide was purchased from Duksan Science. Triethylamine (TEA) and deoxyribonucleic acid sodium salt from salmon sperm testes were purchased from Sigma–Aldrich. Acetone, chloroform, dichloromethane (DCM), methyl alcohol (MeOH), and tetrahydrofuran (THF) were purchased from Samchun Chemicals. All chemicals were used as received without further purification.

### Sample Preparation—Synthesis of CB‐Br, **1**

To a 250 mL round bottom flask equipped with a stir bar were added 4‐cyano‐4′‐hydroxybiphenyl (10.25 mmol, 1 equiv), 1,10‐dibromodecane (32.81 mmol, 3.2 equiv), and acetone (30 mL) followed by potassium carbonate (30.76 mmol, 3 equiv) and trace amount of potassium iodide. The resulting mixture was refluxed at 60 °C for 30 h. The solvent was then removed under reduced pressure using a rotary evaporator. The crude product was dissolved in dichloromethane and vacuum‐filtered to remove undissolved starting material. The crude product underwent extraction to isolate the organic layer using dichloromethane and deionized water. After removing the extraction solvent, the crude product was further washed with excess hexane at room temperature followed by vacuum filtration to afford the desired product, 1. ^1^H NMR analysis of CB‐Br is illustrated in Figure S1 (Supporting Information).

### Sample Preparation—Synthesis of CB‐TEA Cationic Surfactant, **2**

To a 250 mL round bottom flask equipped with a stir bar were added 1 (2.178 mmol, 1 equiv), triethylamine (21.78 mmol, 10 equiv), and chloroform (30 mL). The resulting mixture was refluxed at 65 °C for 72 h. The solvent was removed under reduced pressure, and any remaining triethylamine was removed using a vacuum oven with a temperature set at 60 °C overnight. The crude product was dissolved in methanol, and the resulting mixture was vacuum‐filtered to remove unreacted starting materials. The filtrate was collected, and the solvent was removed. The crude product was then dissolved in tetrahydrofuran using sonication followed by solvent removal. The resulting residue was purified by column chromatography (DCM: MeOH in a volume ratio of 10:1). The crude product was then recrystallized from the minimum amount of water to afford the desired product, 2. ^1^H NMR analysis, WAXS analysis,^[^ ^47^ ^]^ DSC curve, and POM images of CB‐TEA^+^ films are illustrated in Figures S2–S5 (Supporting Information).

### Sample Preparation—Synthesis of DNA‐CB Complex, **3**

In a 1000 mL Erlenmeyer flask equipped with a stir bar, a DNA solution (55 mL) with a concentration of 40 mg mL^−1^ was prepared by dissolving DNA in DI water. A cationic surfactant solution (677 mL) was prepared in a separate flask with a concentration of 10.3 mg mL^−1^ by dissolving the cationic surfactant in DI water. Both solutions were combined by slowly pouring the latter solution into the vigorously stirring former solution. After mixing two aqueous solutions, the water‐stable/insoluble DNA‐cationic surfactant complex precipitates out from the aqueous solution. DNA‐cationic surfactant complex was washed with excess DI water and centrifuged repeatedly to remove unreacted DNA and cationic surfactants. The resulting DNA‐cationic surfactant complex was dried under a vacuum, followed by freeze‐drying to remove the remaining water. Finally, the DNA‐CB complex was washed with chloroform to remove any unwashed residues and then vacuum‐dried. DSC curve and POM images of the DNA‐CB complex are illustrated in Figure S6 (Supporting Information). Upon heating above 90 °C, the DNA‐CB complex gradually lost birefringence and eventually disappears. This was attributed to the denaturation which occured at high temperatures. Fourier‐transform infrared (FT–IR), X‐ray photoelectron spectroscopy (XPS), and Elemental analysis (EA) are illustrated in Figures S7–S9 and Table S1 (Supporting Information).

IR Characterization of DNA‐CB Complex, **3**

Compared to DNA and cationic surfactants, the IR spectrum of the DNA‐CB complex emphasizes differences in peaks, including a shift in the wavenumber as well as the appearance of new peaks. The bare DNA peak was assigned based on the previously reported literature. A sharp peak at 1650 cm^−1^ corresponded to N─H, attributed to hydrogen bonding between base pairs. Additionally, a sharp peak at 1240 cm^−1^ was assigned to the O═P─O a stretch of the phosphodiester bond, which was a linkage between two sugars. Likewise, cationic surfactant peaks were confirmed as the following. Sharp peaks 2220 cm^−1^ represent the CN stretching of a nitrile terminal group. Regarding the DNA‐CB complex, peaks at the functional group region and the fingerprint region of DNA and cationic surfactant are both present in the IR spectrum.

EA Characterization of DNA‐CB Complex, **3**

Quantitative analysis of the chemical composition of elements, including carbon, hydrogen, nitrogen, sulfur, and oxygen (CHNS‐O), was achieved. In the x‐axis, the N/P ratio stands for quaternary amine (N) of cationic surfactant to phosphate (P) of DNA ratio. The N/P ratio of less than 1 indicated that the reaction did not proceed in a stoichiometric ratio of 1:1, rather DNA was in excess. The Y‐axis represented changes in the elemental composition of the DNA‐CB complex as the reaction proceeds. CB‐TEA, which were primarily composed of carbon and hydrogen, sequentially bond to phosphoric acid in DNA, which was initially occupied by the sodium counterion. The replacement occured in a stoichiometric ratio of 1:1 and continues until saturation. Accordingly, there was an overall upsurge in the carbon and hydrogen atom mol%, whereas other elements demonstrated a decrease in atom mol%. Based on EA data, the reaction demonstrated more than 90% complexation.

XPS Characterization of DNA‐CB Complex, **3**

X‐ray Photoelectron Spectroscopy (XPS) analysis was conducted to verify the synthesis of DNA‐CB complex, which was formed by combining DNA‐Na and the cationic surfactant CB‐TEA⁺. For DNA‐Na, the Na 1s peak observed at 1072.1 eV represents the cations, sodium ions, in bare DNA. The N 1s spectrum shows two distinct peaks at 398.5 and 400 eV, corresponding to the nitrogen in the ═NH and ─NH2 groups in the DNA bases, respectively. The P 2p peak at 133 eV reflects the phosphate (─PO4^3^⁻) groups in the DNA backbone. In the case of CB‐TEA⁺, the Na 1s signal was absent since sodium ions were not present. The N 1s spectrum displays peaks at 397.3 and 400 eV, which were attributed to the C═N bonds from the imine groups and the quaternary ammonium groups (N⁺‐R₄), respectively. Similarly, the P 2p signal was absent since there was no phosphorus atom in CB‐TEA⁺. For the DNA‐CB complex, the absence of a Na 1s signal was indicative of the replacement of sodium ions by CB‐TEA⁺. The N 1s spectrum of DNA‐CB showed peaks consistent with the nitrogen in DNA bases and the quaternary ammonium groups (N⁺‐R₄) from CB‐TEA⁺, confirming the incorporation of the cationic surfactant. P 2p spectrum showed the characteristic phosphate peak, verifying the preservation of the DNA backbone structure in the final complex.

### Sample Preparation—Fabrication of DMSO and n‐PrOH Gel, **4**

DNA‐CB complex was immersed in an organic solvent (1 mL), thereby inducing the fabrication of organogels. The solid content, the DNA‐CB complex, ranged from 10 w/v%–50 w/v%. Conversion into wt% yields the following values: 10 w/v% (11 wt%), 20 w/v% (20 wt%), 30 w/v% (27 wt%), 30 w/v% (27 wt%), 40 w/v% (33 wt%), and 50 w/v% (38 wt%).

### Tensile Test

n‐PrOH gel with 50 w/v% (38 wt%) was prepared for tensile test. n‐PrOH gel was fabricated in a dog‐bone shape using a polydimethylsiloxane (PDMS) mold. Each end of the dog‐bone specimen was firmly secured using screws to prevent movement during tensile testing. Next, tensile testing was performed at a constant strain rate of 60 mm min^−1^ using the tensile stress testing stage (Linkam TST350, Tadworth, UK), with force measurements recorded via LINK software. Simultaneously, the orientation changes were also recorded using POM.

### Shear Rheometer

The rheological behavior of organogels was measured using a rheometer with an 8 mm parallel plate (ARES‐G2, TA Instruments, USA). The advanced Peltier system accessory, combined with the 8 mm parallel plate, was used as a bottom plate to accurately adjust the temperature by bottom‐heating or air/coolant‐circulating. The organogels were shaped into disks with a diameter of 8 mm and a thickness of 5 mm and then sandwiched between the plates for rheological measurements. Storage and loss modulus were obtained in the frequency range of 0.016–16 Hz at a fixed strain amplitude of 0.05 at 25 °C. The Z‐axis pull test was performed at a constant strain rate of 0.6 mm^−1^ s^−1^ and 25 °C.

### Dye Adsorption Test

Crystal violet dye was dissolved in DCM at a concentration of 12.5 ppm. Then, the fabricated aerogel was added to the cuvette. Immediately, the cuvette was sealed with a cap, followed by sealing with parafilm to prevent solvent evaporation. Absorbance was measured every hour using a UV–vis spectrometer.

### Characterization

Fourier transform infrared (FT–IR) spectra were measured at room temperature using an FT–IR spectrometer (Thermo Fisher Scientific, Nicolet iN10MX). Scans were taken over a range of 4000–400 cm^−1^. Proton nuclear magnetic resonance (^1^H NMR) spectra were recorded on Bruker AVNEO‐400 (400 MHz) and Bruker AV‐500 (500 MHz) at room temperature. Chemical shifts were reported in parts per million (ppm). X‐ray photoelectron spectroscopy (XPS) spectra were measured using an X‐ray photoelectron spectrometer (NEXSA‐G2, Thermo Fisher Scientific) with Al Kα as an X‐ray source. Elemental analysis (EA) was performed using an elemental analyzer (FlashSmart, Thermo Fisher Scientific). Optical images of DNA‐CB organogel were observed with a POM (Nikon, Eclipse LV100POL) with a multicolor charge‐coupled device camera (Nikon, DS‐Ri1). Field‐emission scanning electron microscope (SEM) images were obtained using SU8230 (Hitachi). Transmission wide‐angle X‐ray scattering (TR‐WAXS) experiments for solution samples were conducted at the 9A U‐SAXS beamline in the Pohang Accelerator Laboratory (PAL). The rheological behavior of organogels was measured with a rotational rheometer (Ares‐G2, TA Instruments) at the Chemical Materials Solutions Center at the Korea Research Institute of Chemical Technology (KRICT). The aerogel was fabricated using a critical point dryer (EM CPD300, Leica). The absorbance was measured by a UV–vis–NIR spectrometer (UV‐3600, Shimadzu).

## Conflict of Interest

The authors declare no conflict of interest.

## Supporting information



Supporting Information

Supplemental Movie 1

Supplemental Movie 2

Supplemental Movie 3

## Data Availability

The data that support the findings of this study are available in the supplementary material of this article.

## References

[1] Y. H. Roh , R. C. H. Ruiz , S. Peng , J. B. Lee , D. Luo , Chem. Soc. Rev. 2011, 40, 5730. 21858293 10.1039/c1cs15162b

[2] C. R. Laramy , M. N. O'Brien , C. A. Mirkin , Nat. Rev. Mater. 2019, 4, 201.

[3] M. Nakata , G. Zanchetta , B. D. Chapman , C. D. Jones , J. O. Cross , R. Pindak , T. Bellini , N. A. Clark , Science 2007, 318, 1276. 18033877 10.1126/science.1143826

[4] F. Livolant , A. M. Levelut , J. Doucet , J. P. Benoit , Nature 1989, 339, 724. 2739716 10.1038/339724a0

[5] S. M. Park , D. K. Yoon , Mater. Horiz. 2024, 11, 1843. 38375871 10.1039/d3mh01585h

[6] J. Kim , H. Yeon , H.‐R. Choi , S. M. Park , C.‐H. Huh , K. C. Choi , D. K. Yoon , Adv. Optic. Mater. 2024, 12, 2400702.

[7] S. Lee , H. Moon , J. Kim , S. Ryu , S. M. Park , D. K. Yoon , Adv. Mater. 2024, 36, 2314374. 10.1002/adma.202314374 38490809

[8] S. M. Park , G. Park , D. K. Yoon , Adv. Mater. 2023, 35, 2302135. 10.1002/adma.202302135 37145961

[9] S. M. Park , G. Park , Y. J. Cha , D. K. Yoon , Small 2020, 16, 2002449. 10.1002/smll.202002449 32686286

[10] Y. J. Cha , S. M. Park , R. You , H. Kim , D. K. Yoon , Nat Commun 2019, 10, 2512. 31175307 10.1038/s41467-019-10540-2 PMC6555807

[11] Y. J. Cha , D. S. Kim , D. K. Yoon , Adv. Funct. Mater. 2017, 27, 1703790.

[12] Y. J. Cha , D. K. Yoon , Adv. Mater. 2017, 29, 1604247. 10.1002/adma.201604247 27862385

[13] Y. J. Cha , M.‐J. Kim , K. Oh , D. K. Yoon , J. Inf. Disp. 2015, 16, 129.

[14] Y. J. Cha , M.‐J. Kim , K. Oh , D. K. Yoon , ACS Appl. Mater. Interfaces 2015, 7, 13627. 26066312 10.1021/acsami.5b03321

[15] J. Y. Kim , S. Choi , G. P. , M. Kim , J. S. Myung , W. J. Choi , S. M. Park , D. K. Yoon , ACS Nano 2023, 17, 22778. 37947399 10.1021/acsnano.3c07493

[16] H. Kominami , K. Kobayashi , H. Yamada , Sci. Rep. 2019, 9, 6851. 31048715 10.1038/s41598-019-42394-5 PMC6497900

[17] J. Gačanin , C. V. Synatschke , T. Weil , Adv. Funct. Mater. 2020, 30, 1906253.

[18] F. Li , J. Tang , J. Geng , D. Luo , D. Yang , Prog. Polym. Sci. 2019, 98, 101163.

[19] H. Qi , M. Ghodousi , Y. Du , C. Grun , H. Bae , P. Yin , A. Khademhosseini , Nat. Commun. 2013, 4, 2275. 24013352 10.1038/ncomms3275 PMC3768014

[20] M. Shin , J. H. Ryu , J. P. Park , K. Kim , J. W. Yang , H. Lee , Adv. Funct. Mater. 2015, 25, 1270.

[21] F. Chen , Y. He , Z. Li , B. Xu , Q. Ye , X. Li , Z. Ma , W. Song , Y. Zhang , Int. J. Pharm. 2021, 606, 120938. 34310955 10.1016/j.ijpharm.2021.120938

[22] Y. H. Kim , K. Lee , S. Li , Chem. Mater. 2021, 33, 7923.

[23] C. Ma , A. Malessa , A. J. Boersma , K. Liu , A. Herrmann , Adv. Mater. 2020, 32, 1905309. 10.1002/adma.201905309 31943419

[24] Y. H. Kim , N. Jeon , S. Park , S. Q. Choi , E. Lee , S. Li , ACS Macro Lett. 2024, 13, 528. 38629344 10.1021/acsmacrolett.4c00028

[25] K. Liu , L. Zheng , C. Ma , R. Göstl , A. Herrmann , Chem. Soc. Rev. 2017, 46, 5147. 28686247 10.1039/c7cs00165g

[26] P. Smith , R. M. Lynden‐Bell , W. Smith , Phys. Chem. Chem. Phys. 2000, 2, 1305.

[27] M. A. Kuzina , D. D. Kartsev , A. V. Stratonovich , P. A. Levkin , Adv. Funct. Mater. 2023, 33, 2301421.

[28] H. Gao , Z. Zhao , Y. Cai , J. Zhou , W. Hua , L. Chen , L. Wang , J. Zhang , D. Han , M. Liu , L. Jiang , Nat. Commun. 2017, 8, 15911. 28639615 10.1038/ncomms15911 PMC5489716

[29] X. Yao , S. Wu , L. Chen , J. Ju , Z. Gu , M. Liu , J. Wang , L. Jiang , Angew. Chem. 2015, 127, 9103. 10.1002/anie.201503031 PMC5033071 26083324

[30] Q. Liu , I. I. Smalyukh , Sci. Adv. 2017, 3, e1700981. 28835927 10.1126/sciadv.1700981 PMC5562421

[31] Z. Meng , Q. Liu , Y. Zhang , J. Sun , C. Yang , H. Li , M. Loznik , R. Göstl , D. Chen , F. Wang , N. A. Clark , H. Zhang , A. Herrmann , K. Liu , Adv. Mater. 2022, 34, 2106208. 10.1002/adma.202106208 34734442

[32] K. T. , Y. Okahata , J. Am. Chem. Soc. 1996, 118, 10679.

[33] S. Otarbayeva , D. Berillo , Gels 2024, 10, 753. 39590109 10.3390/gels10110753 PMC11593573

[34] F. Livolant , Chromosoma 1978, 68, 45. 567570 10.1007/BF00330371

[35] R. Dąbrowski , P. Kula , J. Herman , Crystals 2013, 3, 443.

[36] J.‐H. Lee , S. Oh , I.‐S. Jeong , Y. J. Lee , M. C. Kim , J. S. Park , K. Hyun , T. H. Ware , S.‐K. Ahn , Sci. Adv. 2025, 11, eadt7613. 39823321 10.1126/sciadv.adt7613 PMC11740944

[37] S. H. Back , J. H. Park , C. Cui , D. J. Ahn , Nat. Commun. 2016, 7, 10234. 26725969 10.1038/ncomms10234 PMC4725759

[38] M. J. Han , M. McBride , B. Risteen , G. Zhang , B. V. Khau , E. Reichmanis , D. K. Yoon , Chem. Mater. 2020, 32, 688.

[39] H. Furukawa , K. E. Cordova , M. O'Keeffe , O. M. Yaghi , Science 2013, 341, 1230444. 23990564 10.1126/science.1230444

[40] L. S. Xie , G. Skorupskii , M. Dinca , Chem. Rev. 2020, 120, 8536. 32275412 10.1021/acs.chemrev.9b00766 PMC7453401

[41] T. Hong , C. Lee , Y. Bak , G. Park , H. Lee , S. Kang , T.‐H. Bae , D. K. Yoon , J. G. Park , Small 2024, 20, 2309469. 10.1002/smll.202309469 38174621

[42] Y. Bak , G. Park , T. Hong , C. Lee , H. Lee , T.‐H. Bae , J. G. Park , D. K. Yoon , Nano Lett. 2023, 23, 7615. 37527024 10.1021/acs.nanolett.3c02209

[43] J. Han , Y. Guo , H. Wang , K. Zhang , D. Yang , J. Am. Chem. Soc. 2021, 143, 19486. 34775757 10.1021/jacs.1c08888

[44] M. Peydayesh , M. K. Suter , S. Bolisetty , S. Boulos , S. Handschin , L. Nyström , R. Mezzenga , Adv. Mater. 2020, 32, 1907932. 10.1002/adma.201907932 32026524

[45] Y. Li , Z. Liu , S. Wang , F. Kong , ChemistrySelect 2024, 9, 202402801.

[46] T. Zhang , W. Zhang , H. Xi , Q. Li , M. Shen , G. Ying , J. Zhang , Cellulose 2021, 28, 4281.

[47] K. Liu , D. Chen , A. Marcozzi , A. Herrmann , Proc. Natl. Acad. Sci. USA 2014, 111, 18596. 25512508 10.1073/pnas.1421257111 PMC4284556

