# Supplementary material for: Solvent‐Driven Synthesis of DNA‐Based Liquid Crystalline Organogels with Extraordinary Stretchability, Self‐Healing, and Higher‐Order Structural Assembly
Source: Small. 2025 Mar 11;21(16):2500607. doi: 10.1002/smll.202500607 (PMC12019918; doi:10.1002/smll.202500607)
Supplement: Supplementary file 1 — Supporting Information [file SMLL-21-2500607-s001.docx]

Supporting Information

Solvent-Driven Synthesis of DNA-Based Liquid Crystalline Organogels with Extraordinary Stretchability, Self-Healing, and Higher-Order Structural Assembly

Baekman Kim†, Geonhyeong Park†, Geunjung Lee, Juri Kim, Changjae Lee, Jesse G. Park, Mingeun Kim, Jin Suk Myung, Hyungju Ahn, Soon Mo Park, Woo Jin Choi, and Dong Ki Yoon*


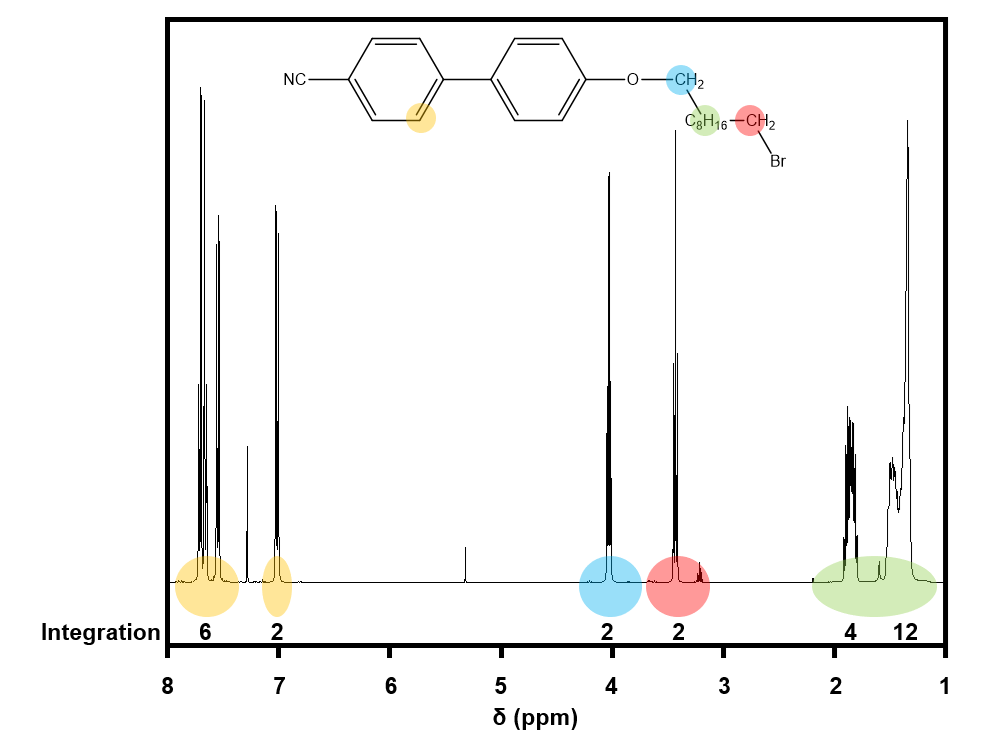


**Figure S1.** Molecular structure and ^1^H NMR analysis of CB-Br. CDCl_3_ was used as a reference. Each proton has been assigned a color accordingly.


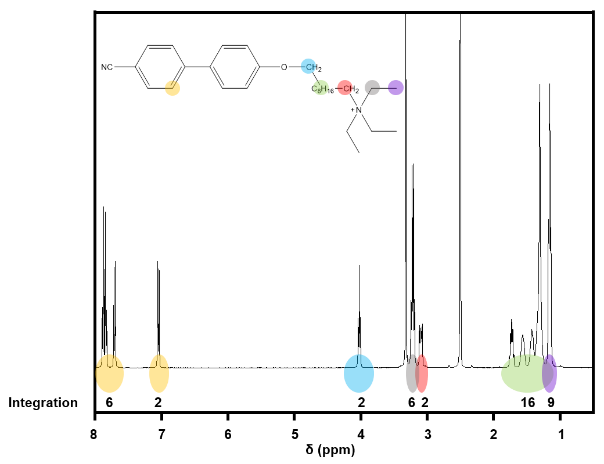


**Figure S2.** Molecular structure and ^1^H NMR analysis of cationic surfactant, CB-TEA^+^. DMSO-d_6_ was used as a reference. Each proton has been assigned a color accordingly.


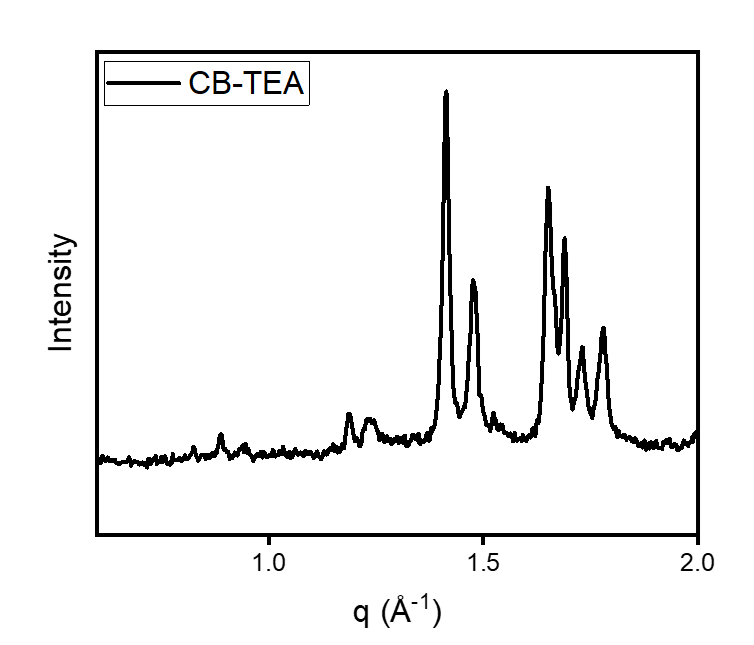


**Figure S3.** Wide-angle X-ray Scattering (WAXS) profile of the CB-TEA surfactant. The result shows distinct diffraction peaks at the q range of 1.4 ~ 1.6 Å⁻¹, which corresponds to a d-spacing of 0.39 ~ 0.45 nm. This value is attributed to the layered packing structure of the CB-TEA surfactant.^[48]^


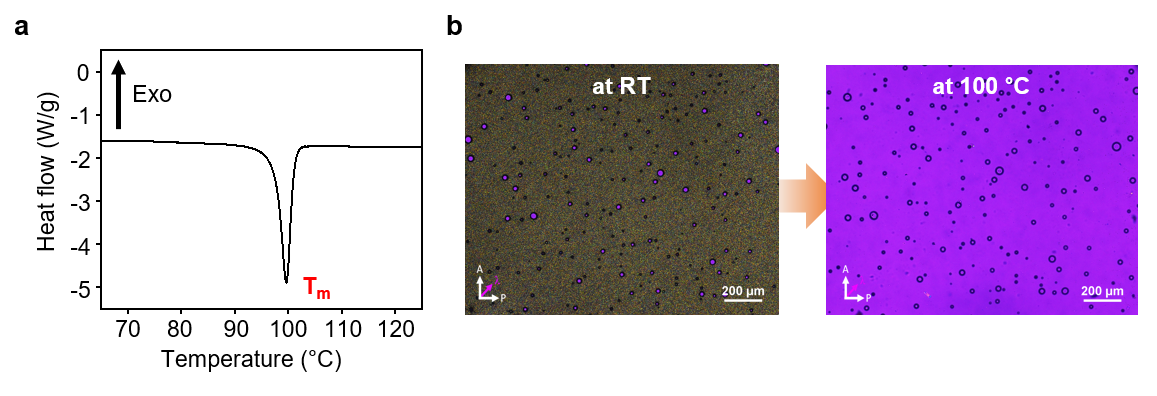
**Figure S4.** a) DSC curve of cationic surfactant, CB-TEA^+^ and b) corresponding POM images.


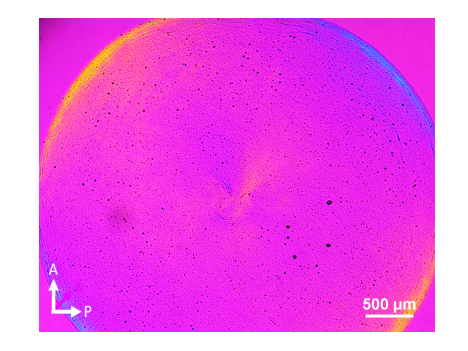


**Figure S5.** POM image of CB-TEA+ film on a glass slide. The scale bar is 500 µm. Blue domains indicate that the director of CB-TEA surfactant is oriented perpendicular to the slow axis of the retardation plate (λ = 530 nm). In contrast, yellow domains represent that CB-TEA surfactant aligns in a parallel direction to the slow axis of the retardation plate.


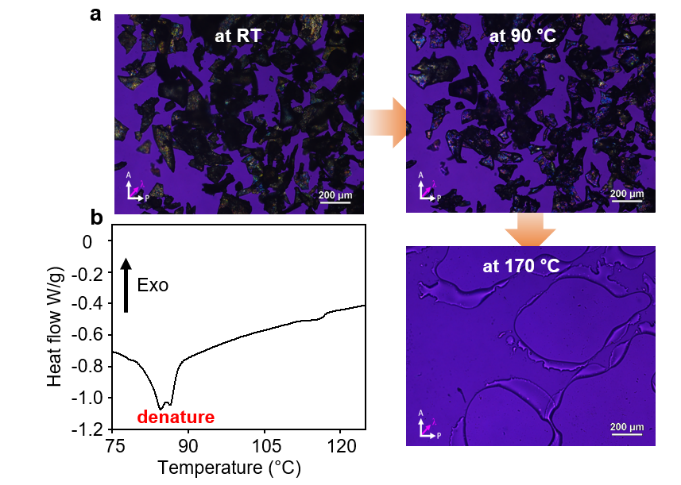


**Figure S6.** a) POM images of DNA-CB complex with increasing temperature and corresponding DSC curve.


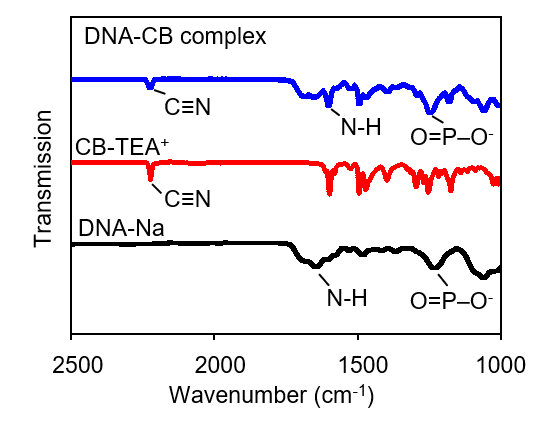


**Figure S7.** Fourier-transform infrared (FT-IR) spectra of synthesized DNA-CB complex (blue), CB-TEA^+^ (red), and DNA-Na (black).


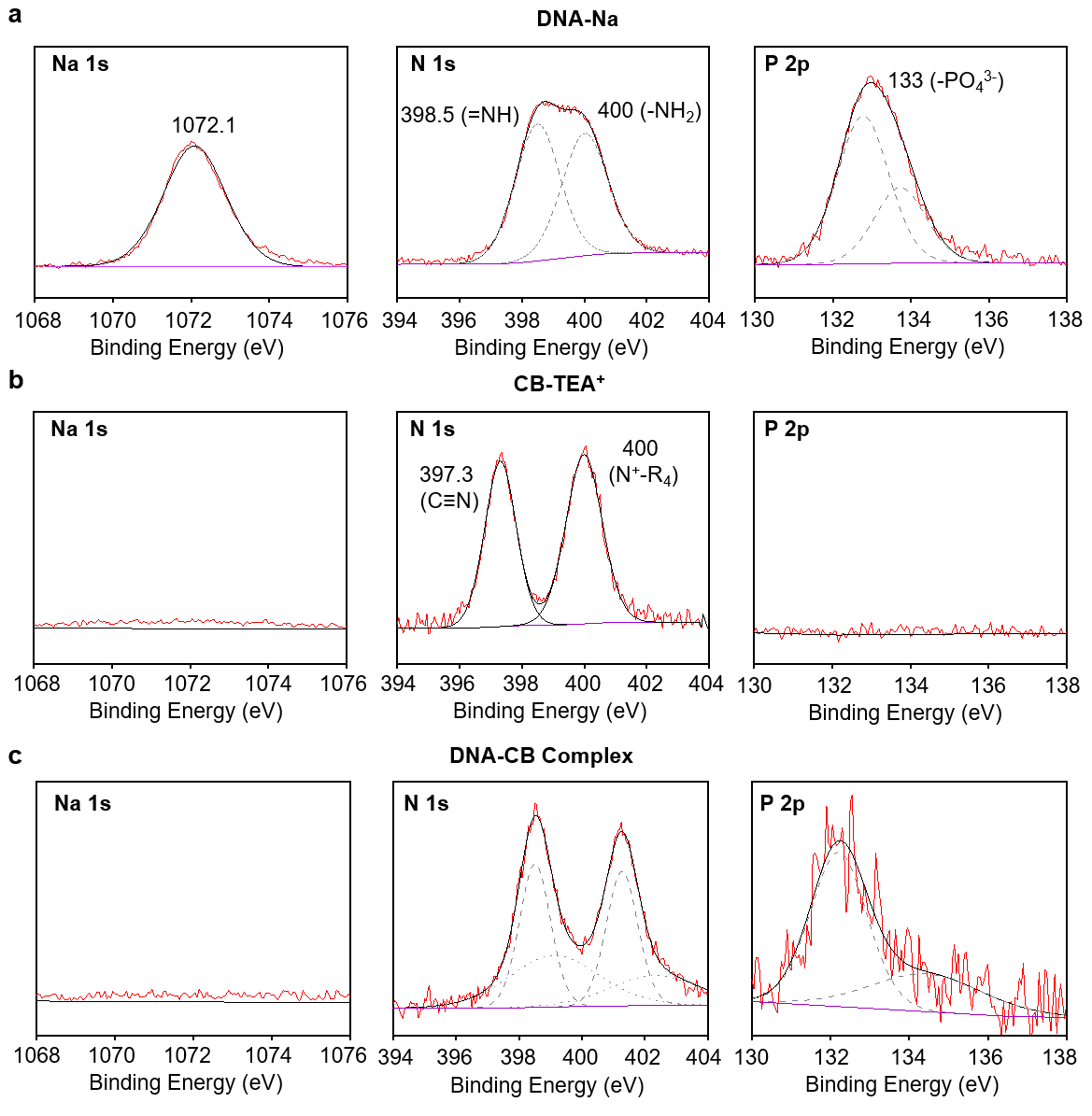
**Figure S8.** XPS analysis of a) DNA-Na, b) synthesized cationic surfactant, CB-TEA^+^, and c) synthesized DNA-CB complex. TiO2 was used as a reference.


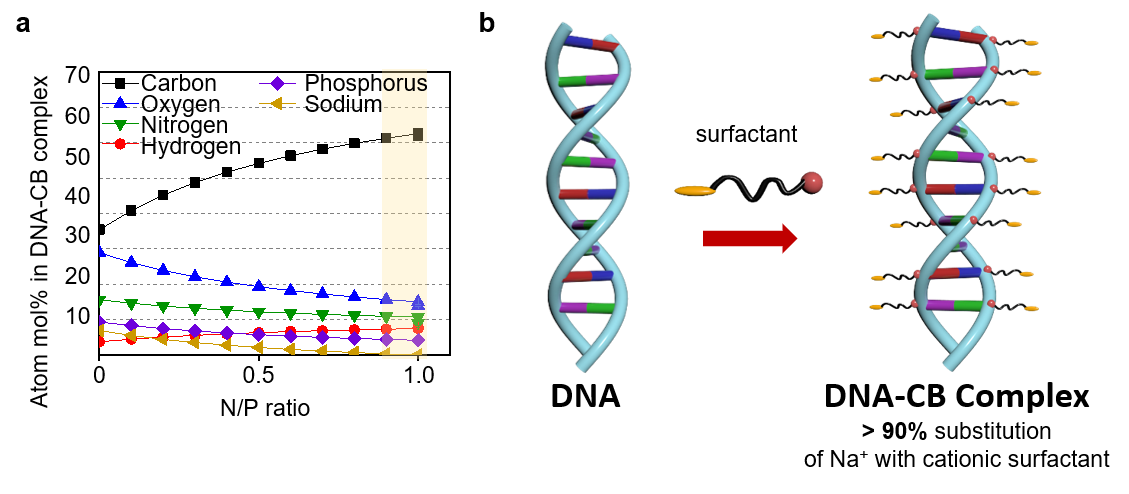


**Figure S9.** a) Elemental Analysis (EA) of synthesized DNA-CB complex.


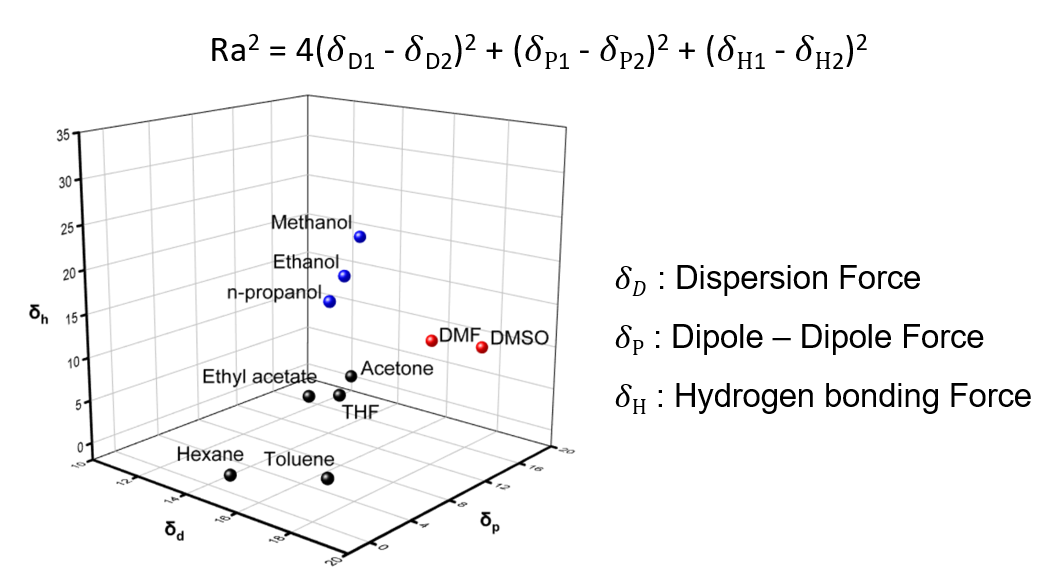


**Figure S10.** Visualization of 3D Hansen solubility parameter space of CB-TEA^+^ surfactants within DNA-CB complex with various solvents. The color of the dot (red, blue, black) indicates solvation, swelling, and no reaction, respectively.


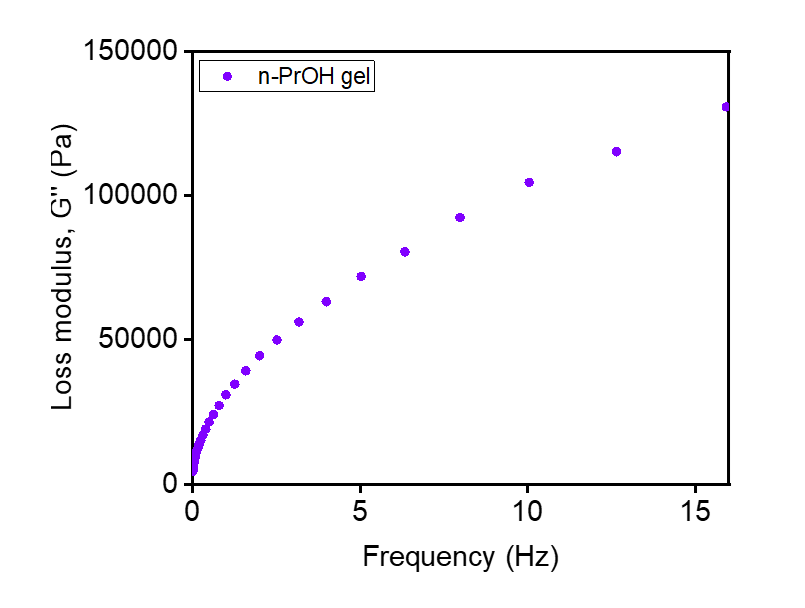


**Figure S11.** Dynamic mechanical analysis of n-PrOH gel’s loss modulus (G’’) by frequency sweep mode using a shear rheometer.


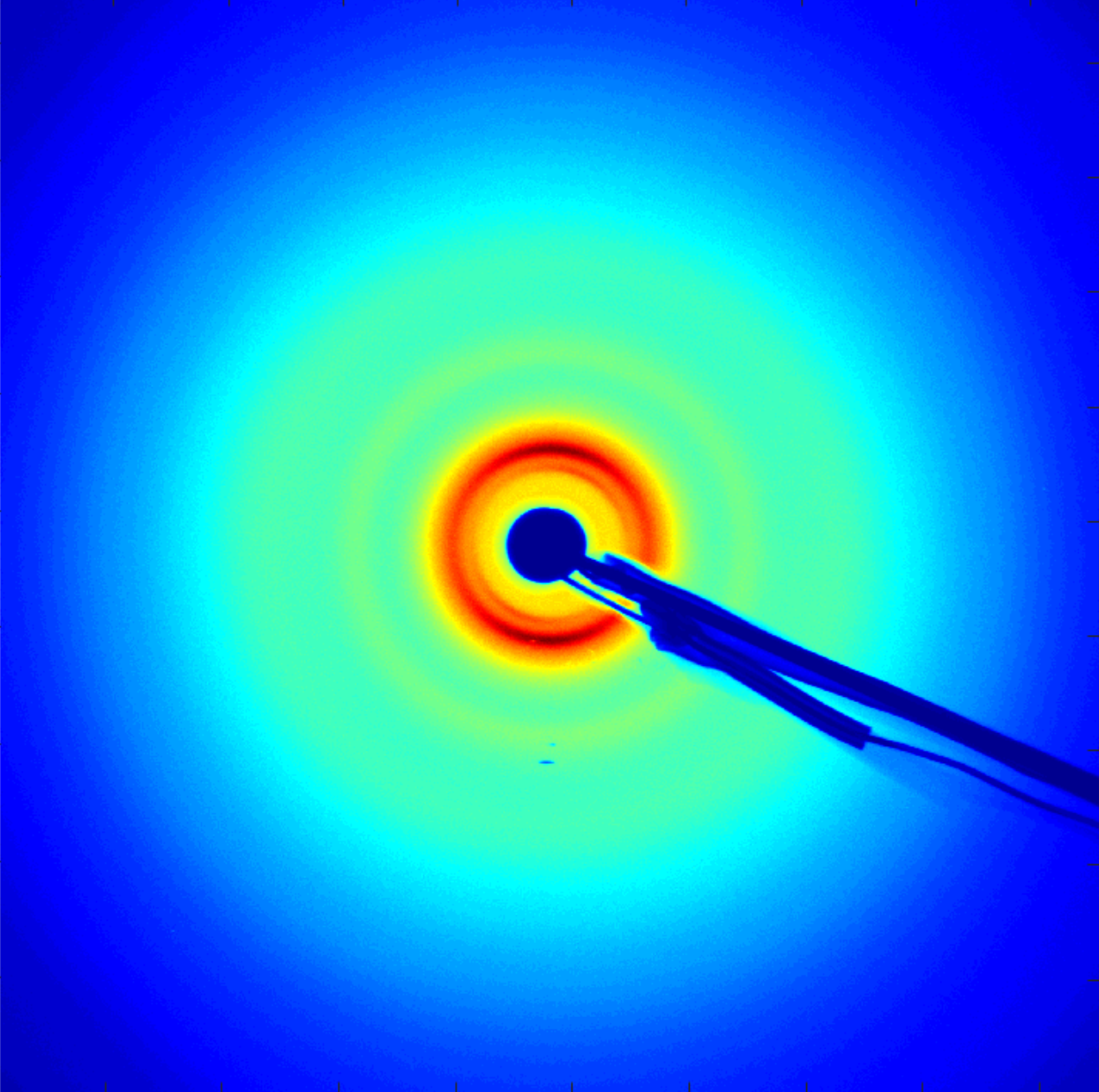

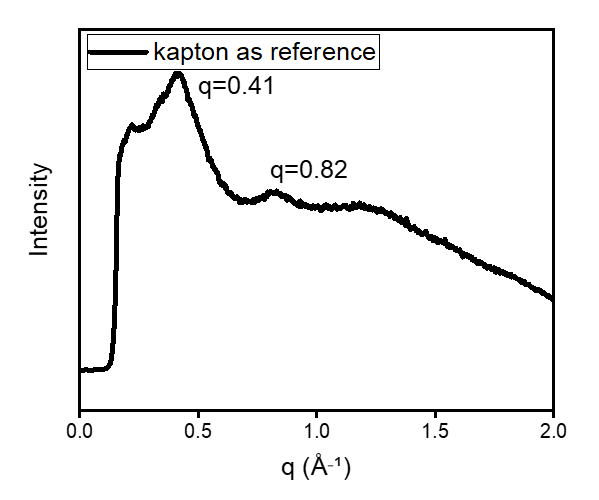


**Figure S12.** Wide-angle X-ray Scattering (WAXS) profile of Kapton tape.


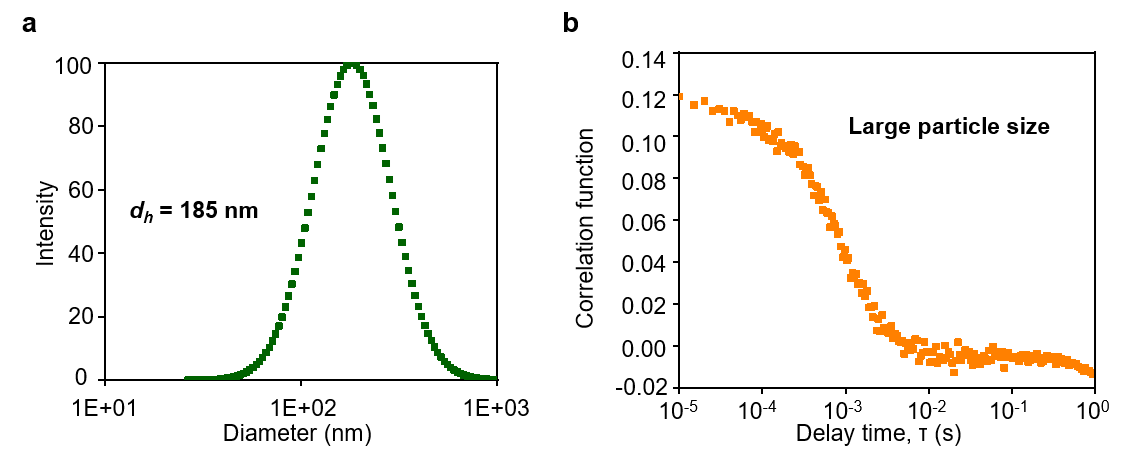


**Figure S13.** DLS analysis of DMSO organogel at a 0.01 mg/ml concentration. a) particle size distribution. b) autocorrelation curve.


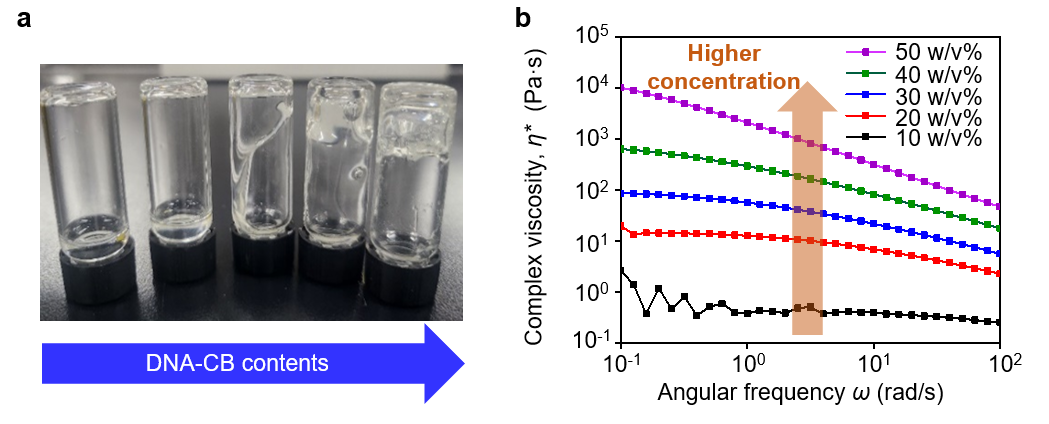


**Figure S14.** Characterization and analysis of fabricated DMSO organogel. a) Photograph illustrating vial inversion test of DMSO organogel with increasing DNA-CB complex contents. b) Frequency-dependent complex viscosity of DMSO organogel. Viscous organogel demonstrates shear thinning behavior.


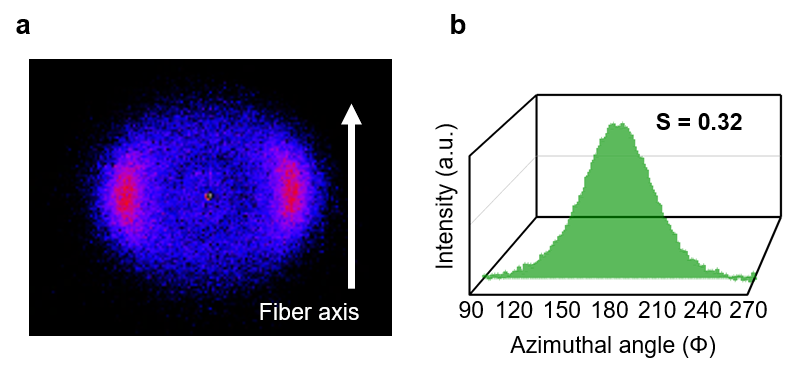


**Figure S15.** Characterization and analysis of fabricated drawn fiber from DMSO organogel. a) 2D X-ray diffraction images. The arrow indicates the direction of the fiber’s orientation. b) Orientation order parameter of drawn fiber from DMSO organogel.


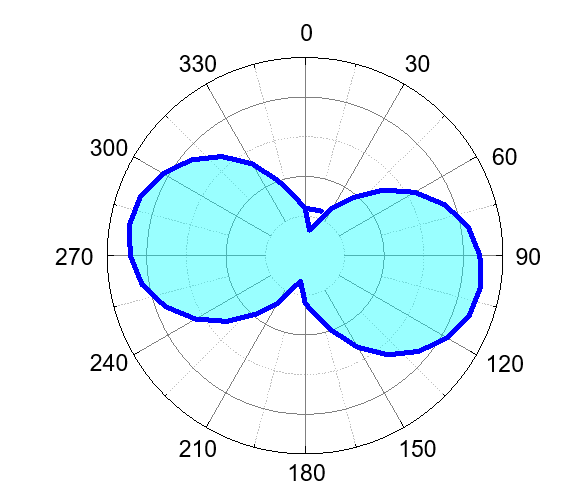


**Figure S16.** Polarized FT-IR of fiber from DMSO organogel. The orientation of the functional group can be deduced by calculating the maximum absorption intensity when the functional group’s vibration axis is parallel to that of polarized IR.


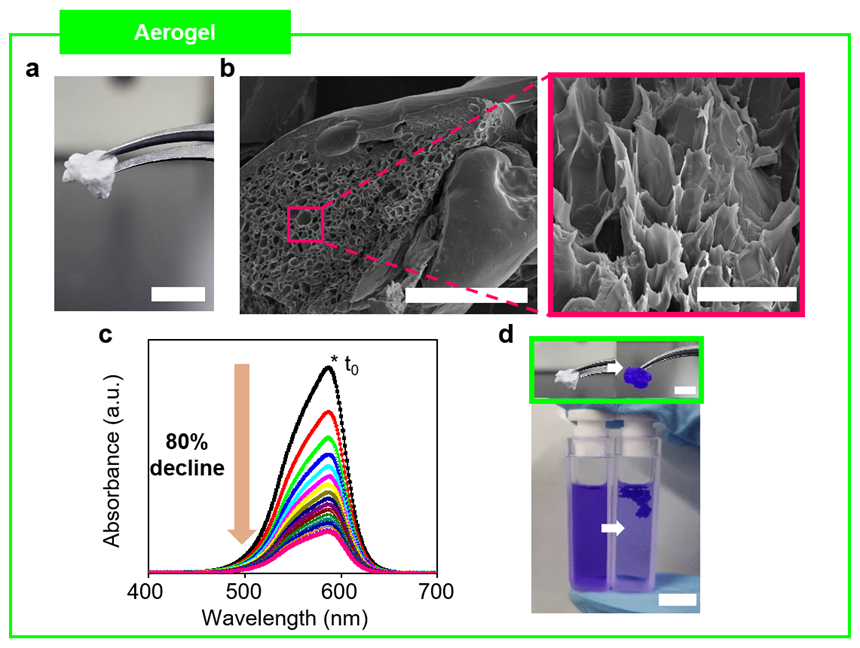


**Figure S17.** Fabrication and characterization of DNA-TEA aerogel via critical point dryer (CPD). a) Photograph of a fabricated aerogel. b) SEM images of a porous aerogel. c) UV-Vis analysis on time-dependent dye adsorption of DNA-TEA aerogel to indicate the initial measurement. Each color indicates an hour. d) Illustration of dye removal by DNA-TEA aerogel. The inset illustrates changes in the aerogel after dye adsorption. Crystal violet was used as a dye. The scale bars are a) cm; b) 100 µm and 10 µm (SEM images); d) 1 cm, respectively.


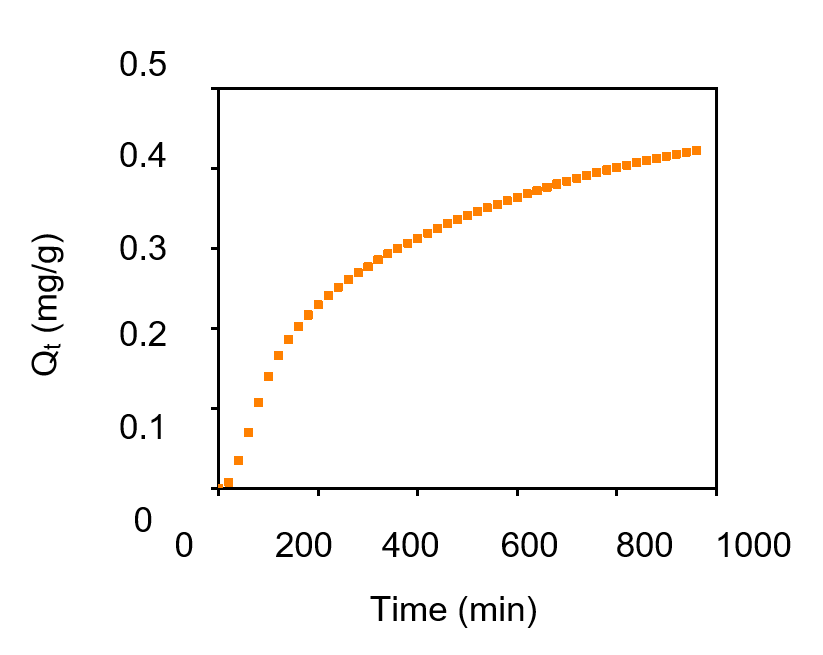


**Figure S18.** Time-dependent dye adsorption capacity of DNA-TEA aerogel.


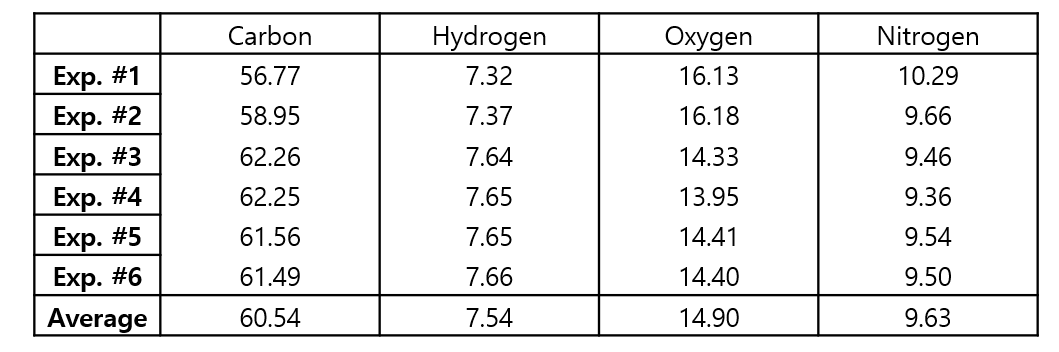


**Table S1.** Experimental result table of Elemental Analysis.
